# AAPM Medical Physics Practice Guideline 12.a: Fluoroscopy dose management

**DOI:** 10.1002/acm2.13526

**Published:** 2022-02-17

**Authors:** Ryan F. Fisher, Kimberly E. Applegate, Lindsey K. Berkowitz, Olav Christianson, Jaydev K. Dave, Lindsay DeWeese, Nichole Harris, Mary Ellen Jafari, A. Kyle Jones, Robert J. Kobistek, Brendan Loughran, Loren Marous, Donald L. Miller, Beth Schueler, Bryan C. Schwarz, Adam Springer, Kevin A. Wunderle

**Affiliations:** ^1^ Department of Radiology The Metro Health System Cleveland Ohio USA; ^2^ Department of Radiology College of Medicine University of Kentucky Lexington Kentucky USA; ^3^ Department of Radiology Maine Medical Center Portland Maine USA; ^4^ Clinical Dose Optimization Service LANDAUER Medical Physics Glenwood Illinois USA; ^5^ Department of Radiology Thomas Jefferson University Philadelphia Pennsylvania USA; ^6^ Department of Diagnostic Radiology Oregon Health & Science University Portland Oregon USA; ^7^ Department of Radiology University Hospitals Cleveland Medical Center Cleveland Ohio USA; ^8^ Department of Radiation Physics & Safety Atlantic Medical System Morristown Morristown New Jersey USA; ^9^ Department of Imaging Physics The University of Texas MD Anderson Cancer Center Houston Texas USA; ^10^ RJK Medical Physics, Inc. Cleveland Ohio USA; ^11^ Clinical Dose Optimization Service™/OPTIMIZE™ Division LANDAUER Medical Physics Glenwood Illinois USA; ^12^ Upstate Medical Physics, P.C. Victor New York USA; ^13^ Center for Devices and Radiological Health U.S. Food and Drug Administration Silver Spring Maryland USA; ^14^ Mayo Clinic Department of Radiology Rochester Minnesota USA; ^15^ Department of Radiology University of Florida Gainesville Florida USA; ^16^ KLS Physics Group, LLC Ruston Louisiana USA; ^17^ Cleveland Clinic Department of Radiology Cleveland Ohio USA

**Keywords:** fluoroscopy, MPPG, practice guideline

1

The American Association of Physicists in Medicine (AAPM) is a nonprofit professional society whose primary purposes are to advance the science, education, and professional practice of medical physics. The AAPM has more than 8,000 members and is the principal organization of medical physicists in the United States.

The AAPM will periodically define new practice guidelines for medical physics practice to help advance the science of medical physics and to improve the quality of service to patients throughout the United States. Existing medical physics practice guidelines will be reviewed for the purpose of revision or renewal, as appropriate, on their fifth anniversary or sooner.

Each medical physics practice guideline represents a policy statement by the AAPM, has undergone a thorough consensus process in which it has been subjected to extensive review, and requires the approval of the Professional Council. The medical physics practice guidelines recognize that the safe and effective use of diagnostic and therapeutic radiology requires specific training, skills, and techniques, as described in each document. Reproduction or modification of the published practice guidelines and technical standards by those entities not providing these services is not authorized.

The following terms are used in the AAPM practice guidelines:

- Must and Must Not: Used to indicate that adherence to the recommendation is considered necessary to conform to this practice guideline. **While must is the term to be used in the guidelines, if an entity that adopts the guideline has shall as the preferred term, the AAPM considers that must and shall have the same meaning**.
- Should and Should Not: Used to indicate a prudent practice to which exceptions may occasionally be made in appropriate circumstances.

## 2 INTRODUCTION

Fluoroscopy equipment is used to observe or guide moving objects such as internal organs, contrast agents, catheters, and guidewires within the body to diagnose and treat disease. Procedure times range from several seconds to multiple hours, and fluoroscopes range from small, mobile C‐arms used to image extremities, to complex single‐ or bi‐plane angiography systems. These more complex fluoroscopes are used to guide performance of fluoroscopically guided interventional (FGI) procedures, and help to provide lifesaving diagnostic and therapeutic services for patients. However, unlike simpler procedures commonly accomplished using general or mobile C‐arm fluoroscopes, long and complex FGI procedures can exceed radiation thresholds for tissue reactions. Proper identification, follow‐up, and management of patients receiving high doses from FGI procedures are essential parts of patient care due to the slowly developing nature of radiation‐induced tissue reactions. Recent standards and requirements from accrediting bodies such as The Joint Commission (TJC) and state regulatory agencies have brought focus to this issue, requiring hospitals to record patient fluoroscopy exam dose indices and to establish committees, policies, and procedures for reviewing those data and providing patient follow‐up as appropriate. These standards are in addition to TJC's updated fluoroscopy sentinel event standard, which requires identification and investigation of severe tissue effects.

Many organizations and societies have provided guidance and resources for managing patient dose, including the National Council on Radiation Protection and Measurements (NCRP), the Conference of Radiation Control Program Directors (CRCPD), the Department of Veterans Affairs, the Society of Interventional Radiology (SIR), and multiple cardiology societies under the umbrella of the American College of Cardiology Task Force on Expert Consensus Decision Pathways.^1–5^ This AAPM practice guideline aims to outline the role of the diagnostic qualified medical physicist (QMP), as defined by AAPM Policy Number PP 1‐J “Definition of A Qualified Medical Physicist,” in practical patient dose management for FGI procedures.^6^ This role includes helping facilities set up policies related to dose management, including pre‐procedure patient consent, intra‐procedure dose index level notification, and post‐procedure follow‐up for potential tissue reactions. Suggestions for methods of complying with TJC standards and various state regulatory requirements for tracking radiation use, setting dose index thresholds, and analyzing dose index data are provided, along with a discussion of the challenges posed by these requirements. The QMP's role in helping facilities comply with TJC's updated “radiation overdose” sentinel event standards by investigating severe tissue reactions is also discussed. Related fluoroscopy topics that may fall under the QMP's oversight, such as operator credentialing and occupational radiation exposure monitoring are briefly discussed.

## 3 TISSUE REACTIONS

Tissue reactions, also known as deterministic effects, are due to radiation effects on populations of cells and are characterized by a threshold dose and an increase in the severity of the reaction with increasing dose.^7^ These reactions are the primary concern for patients undergoing FGI procedures, and will be the focus of this report, with stochastic risks not being addressed. Tissue reactions do not occur at doses below a threshold dose, which the International Commission on Radiological Protection (ICRP) defines as the dose estimated to result in a 1% incidence of the tissue reaction.^8^ Tissue reactions in patients undergoing FGI procedures may involve skin, hair, subcutaneous fat, muscle, the lens of the eye, and sometimes bone.^9^, ^10^ The generally accepted minimum threshold dose for transient skin effects is an absorbed skin dose of approximately 2 Gy, and permanent effects are unlikely below an absorbed skin dose of 5 Gy.^1^, ^11^ Risks for tissue reaction can conservatively be assumed as cumulative when the same skin area has been irradiated for other procedures. Repair of sublethal radiation injury to skin is typically complete within a day of exposure; repopulation of cells can require months.^11^ Tissue reactions may be expressed days to years after exposure, depending on the radiation dose and the tissue affected. Early reactions may be due to inflammation, and may not be noticed by the patient, whereas late reactions are typically due to cell loss. Tissue reactions in the skin range in severity from erythema and transient epilation to dermal necrosis, which can require surgical intervention.^12^ Because of individual variability in radiosensitivity, the radiation dose necessary to produce a specific effect and the time course of the tissue reaction are best thought of as ranges, rather than specific values, as shown in Table 1, reprinted from Balter et al.^11^ Additionally, it should be noted that previously irradiated skin is at a higher risk for developing tissue reactions than areas that have had no prior exposure.

**TABLE 1:** Tissue reactions from single‐delivery radiation dose to skin of the neck, torse, pelvis, buttocks, or arms

	Single‐site					
	acute skin	NCI skin				
	dose range	reaction	Approximate time of onset of effects			
Band	(Gy)^a^	grade	Prompt	Early	Midterm	Long term
A1	0–2	NA	No observable effects expected	No observable effects expected	No observable effects expected	No observable effects expected
A2	2–5	1	Transient erythema	Epilation	Recovery from hair loss	No observable results expected
B	5–10	1–2	Transient erythema	Erythema, epilation	Recovery; at higher doses, prolonged erythema; permanent partial epilation	Recovery; at higher doses, dermal atrophy or induration
C	10–15	2–3	Transient erythema	Erythema, epilation; possible dry or moist desquamation; recovery from desquamation	Prolonged erythema; permanent epilation	Telangiectasia^b^; or induration; skin likely to be weak
D	>15	3–4	Transient erythema; after very high doses, edema and acute ulceration; long‐term surgical intervention likely to be required	Erythema, epilation; moist desquamation	Dermal atrophy; secondary ulceration due to failure of moist desquamation to heal; surgical intervention likely to be required; at higher doses, dermal necrosis, surgical intervention likely to be required	Telangiectasia^b^; dermal atrophy or induration; possible late skin breakdown; wound might be persistent and progress into a deeper lesion; surgical intervention likely to be required

*Note*: Applicable to normal range of patient radiosensitivities in absence of mitigating or aggravating physical or clinical factors. Data do not apply to the skin or the scalp. Dose and time bands are not rigid boundaries. Signs and symptoms are expected to appear earlier as skin dose increases. Prompt, <2 weeks; early, 2–8 weeks; midterm, 6–52 weeks; long term, >40 weeks.

Abbreviations: NA, not applicable; NCI, National Cancer Institute.

^a^ Skin dose referes to actual skin dose (including backscatter). This quantity is not the reference point air kerma described by Food and Drug Administration (21 CFR § 1020.32 [2008]) or International Electrotechnical Commission. Skin dosimetry is unlikely to be more accurate than ±50%.

^b^ Refers to radiation‐induced telangiectasia. Telangiectasia associated with area of initial moist desquamation or healing of ulceration may be present earlier.

*Source*: Reproduced from Balter et al.,^11^ Tissue reactions from radiation doses to the skin.

## 4 AVAILABLE DOSE INDICES

Predicting the likelihood of radiation‐induced effects from FGI procedures requires an estimation of the patient's radiation dose. Four measurable radiation dose indices exist to assist with this estimation: fluoroscopy time, cumulative air kerma (*K*_a,r_ or CAK), air kerma–area product (*P*_KA_, also commonly written as KAP or dose–area product/DAP), and peak skin dose (PSD). Effective dose is not suitable for assessing the likelihood of tissue effects. The availability and displayed units of each dose index vary depending on equipment type, manufacturer, and equipment age, and each differs in clinical utility and application.

*Fluoroscopy time* is the most widely available index; however, it is also the least useful in terms of predicting potential tissue effects. Fluoroscopy time alone is inadequate to estimate patient dose. It does not consider fluoroscopic dose rate, and dose estimates that rely solely on fluoroscopy time can vary widely, as acquisitions (e.g., cine, Digital Subtraction Angiography (DSA)), which can contribute substantial dose, are not included in the measurement.^13^ If additional dose rate and dose per image data are available for the specific mode of operation used, using the number of fluorographic images from a procedure, along with fluoroscopy time, can improve estimations of patient dose. However, while fluoroscopy time is suboptimal for assessing radiation dose, it can be useful for other purposes (e.g., a surrogate for procedure complexity and comparing practice among operators).

*CAK* (*K*_a,r_) is required to be displayed on all International Electrotechnical Commission (IEC) compliant interventional fluoroscopes and all fluoroscopes sold in the United States since June 2006.^4^, ^14^, ^15^ *K*_a,r_ is the cumulative kerma for a fluoroscopic procedure, including fluoroscopic and acquisition modes of operation, measured in air at a specific reference point relative to the X‐ray source. For isocentric C‐arms, the IEC definition for the reference point is along the central ray of the X‐ray beam, 15 cm from the isocenter toward the X‐ray tube, though manufacturers can use a different reference point if they choose. The specific reference point used by a piece of equipment is defined in the operator's manual. *K*_a,r_ is often used as a surrogate for the patient's PSD; however, potentially labor‐intensive corrections and calculations are required if a more accurate estimate of PSD is needed. These corrections include backscatter, table and pad attenuation, displayed dose index accuracy, and tissue‐to‐air ratio. Additionally, due to beam geometry, gantry angulation, table height, and patient size, the reference point may not coincide with the entrance skin surface. This results in a tendency for *K*_a,r_ to overestimate PSD. Despite these shortcomings, *K*_a,r_ is generally considered a practical surrogate for skin dose.^2^ *K*_a,r_ is almost universally reported in units of milligray (mGy) in modern fluoroscopes.

*KAP* (*P*_KA_), sometimes called DAP, is the product of air kerma and the geometric area irradiated in the same plane orthogonal to the propagation of the X‐ray beam. *P*_KA_ displays are widely available on interventional fluoroscopes and commonly available on many modern mobile C‐arms and conventional diagnostic fluoroscopy equipment. Unlike *K*_a,r_, *P*_KA_ is independent of distance from the focal spot, because the irradiated area increases proportionally to the decreased radiation intensity as distance increases. Therefore, small doses to a large area and large doses to a small area could give equal *P*_KA_ values. For this reason, *P*_KA_ is considered a poor indicator of skin dose and radiation‐induced tissue effects. Because *P*_KA_ represents the total energy deposited in the patient, it is better correlated to stochastic risk as compared with *K*_a,r_. Additionally, a lack of standardized units for displayed *P*_KA_ values on fluoroscopic equipment can make the practical implementation of clinical thresholds difficult. *P*_KA_ displayed units of uGy × m^2^, mGy × cm^2^, cGy × cm^2^, and Gy × cm^2^ are all in use.

*PSD* indicators, with real‐time dose mapping displays, are the least common dose index available at present but are becoming increasingly common on modern FGI equipment. They allow the operator to visualize the three‐dimensional skin dose distribution, potentially preventing tissue reactions. Freestanding software, independent of the FGI equipment, is also available that can estimate PSD based on data provided in the radiation dose structured report (RDSR). Real‐time PSD information provides the greatest clinical utility for predicting the likelihood of tissue reactions because it provides estimates of the highest skin dose and its location using information on patient position, X‐ray field size, and beam angulation during a procedure. Freestanding software that reconstructs PSD from RDSR information also has clinical utility but lacks direct feedback to the operator during a procedure. It is important to be aware of manufacturer‐specific approaches to PSD estimates and their level of sophistication with regard to inclusion of correction factors such as backscatter, table and table pad attenuation, and patient anatomical representation. Because of these differences in approach, it is possible that fluoroscopes from different manufacturers, or even different versions of the same manufacturer's software, could provide different PSD values given identical RDSRs. A review of various PSD software options was presented by Malchair et al.^16^ At present, real‐time PSD estimates are limited to FGI fluoroscopes and are not found on mobile C‐arms or general fluoroscopic equipment. PSD is commonly reported in units of milligray (mGy).

In setting up a patient fluoroscopy dose management program, the QMP will need to survey the dose indices available on the imaging equipment in a facility or larger healthcare system. The availability of these indices will determine the dose index used to set thresholds for further action. Some dose indices may be available but require equipment configuration in order to be displayed. For older equipment that does not display *K*_a,r_ or *P*_KA_, aftermarket meters can provide this capability. Ideally, one would use PSD, the dose index that best correlates with potential tissue injury, but its limited availability and the variability in assumptions and corrections made by PSD algorithms complicate its use at the present time. Due to its standard definition, ubiquitous implementation, and reasonable correlation with PSD, it is the recommendation of this group that CAK (*K*_a,r_) be the primary dose index used in setting fluoroscopic threshold dose levels for notification and patient follow‐up.

## 5 FGI POLICY

The following sections broadly describe the policies that facilities must have in place, regardless of regulatory or accreditation requirements, to increase stakeholder awareness and to help prevent, identify, and properly care for patients with tissue reactions due to high‐dose FGI procedures. These policies are broken into three sections:

1. Pre‐procedure screening and consent of the patient.
2. Intra‐procedure monitoring and notification of patient dose index level to the team.
3. Post‐procedural patient follow‐up above threshold levels.

While QMPs are not typically involved in the day‐to‐day implementation of these policies and procedures, their expertise is critical in development of the policies. Additionally, the QMP can be consulted when specific questions arise and may be called upon for PSD or other dose estimates.

### 5.1 Pre‐procedure consent for risk of skin injury

Each healthcare facility must create a policy for obtaining a radiation‐specific consent from patients prior to FGI procedures where patients are given information regarding the risks of radiogenic tissue effects. Whenever possible, this policy should be standardized across all departments utilizing FGI equipment across the entire organization. Local laws may dictate whether consent can be verbal or must be written. A facility may choose to obtain this consent before all procedures performed in FGI suites or only before a subset of procedures classified as potentially high dose. The QMP can aid in reviewing facility data to determine which procedures require consent. NCRP Report No. 168 suggests classifying procedures as “potentially high radiation dose procedures” if more than 5% of procedures result in *K*_a,r_ exceeding 3 Gy.^1^ Obtaining a radiation consent for only a subset of procedures removes a clinical step prior to procedures where tissue effects are highly unlikely. However, it may complicate policy and process by requiring someone to create and keep updated a list of procedures in radiology, cardiology, surgery, and other specialties requiring radiation consent.

An example of simple radiation consent language, adapted from the SIR's guidelines for patient dose management, can be found in Appendix A.^17^ Additional example consent language can be found in NCRP Report Nos. 168 and 185.^1^, ^18^ Any such document should be reviewed by appropriate clinical and legal teams prior to implementation. Key elements of consent language should include a description of the use of X‐rays in fluoroscopy, the possible tissue effects resulting from prolonged exposure to X‐rays, the typical time delays for these effects to occur, and the proposed action(s) for the patient and/or caregiver if any effect is observed.

### 5.2 Pre‐procedure screening

In addition to screening for potential medical issues or pregnancy, patients who will undergo potentially high‐dose FGI procedures should be screened to determine if they are at higher risk for tissue effects. The result of pre‐procedure patient screening is then conveyed to the performing physician. Patients at higher risk include those who have had recent high‐dose fluoroscopy procedures or a history of radiation therapy to the same skin area, collagen vascular disease, or certain genetic disorders that affect DNA repair, which are further detailed in Ref.^11^ High body mass index (BMI) is also a risk factor since the greater amount of tissue increases the amount of radiation needed to yield an adequate image and can result in the skin being closer to the X‐ray source.^19^

### 5.3 Intra‐procedure monitoring and notifications

Intra‐procedural notifications regarding radiation dose levels allow the performing physician to gauge the benefit–risk ratio at each stage of an FGI procedure. All FGI procedures should be justified, that is, offer a clinical benefit to the patient greater than the potential risks, which include radiation tissue reactions.^1^ This benefit–risk ratio is considered by the physician when initially deciding whether to perform a procedure, and later, while the procedure is in progress. For the benefit–risk ratio to be meaningful, the physician needs to have an accurate understanding of radiation risk, the likelihood of tissue injury, and the associated dose–response relationship.^11^ The QMP, as the subject matter expert in this area, can provide radiation protection planning knowledge to the team to help ensure that the benefit–risk ratio is formulated correctly. The QMP must also understand the magnitude of radiation risk compared to other procedural risks, which are often much greater than the risk of tissue reaction. The risk of a radiation effect is typically much smaller than other procedural risks, such as bleeding, infection, and organ damage. However, the benefit–risk ratio is not static, and may change during the procedure. This analysis should be performed by a well‐informed operator and should be evaluated throughout the procedure. Procedures should rarely, if ever, be stopped solely due to radiation dose. If all or most progress made during the procedure is lost if the procedure is stopped (e.g., navigation of catheter to a very difficult site, or risk of developing collateral vasculature in the interval), any risks already incurred will have been for no benefit to the patient.^1^ When appropriate clinically, very complex procedures may be planned in a staged fashion, with multiple sessions separated by 8–10 weeks, to fractionate the dose to the skin and reduce the likelihood of tissue reactions.

The concept and implementation of radiation dose notification levels is simple. The same concept has been applied to other potentially toxic agents, such as iodine contrast or medications.^1^ The implementation of notification levels requires the entire procedure team to work together. For example, the radiologic technologist, who is a local expert on the operation of fluoroscopy equipment, may be the one who calls out when the notification level is reached and documents this action in the procedure record. The nurse may then document the same information in the electronic medical record, while the operator or performing physician pauses for a moment to consider their radiation management strategy and the current benefit–risk pace of the procedure.

The essential elements of an intra‐procedural dose notification are:

1. Verbal notification to the operator regarding the current notification number and the magnitude of the dose index, for example, “This is the third notification. The current reference air kerma is 5000 mGy.”
2. Consideration of a procedural pause to evaluate setup and the benefit–risk pace of the procedure, if such a pause will not interfere with the conduct of the procedure.
3. Documentation of the notification level, that the operator was notified, that the setup and benefit–risk ratio of the procedure were evaluated, and any specific actions taken by the operator.

The notification should be delivered during a natural pause during the procedure, if possible, and should never interrupt a critical phase of the procedure. Re‐consideration of radiation dose management strategy after a notification may include specific actions including, but not limited to:

1. Adjustment of the patient table height. Sometimes it is set within the optimal range at the beginning of the procedure, but may be lowered during the procedure, for example, to isocenter the patient for volumetric imaging, after which the table is not moved back to the original height.
2. Elimination of a large air gap by lowering the image receptor.
3. Evaluation of X‐ray beam collimation and gantry angle.
4. Evaluation of currently selected organ program (aka imaging protocol) for aspects such as dose rate setting, pulse rates, and DSA frame rate.

Traditional radiation management practice often suggests that the angle of the gantry be varied during a procedure to “spread” radiation dose across the skin. While this strategy can be useful in specific circumstances as a prophylactic technique, it can be detrimental to radiation management and greatly increase both the skin dose rate and the PSD, especially in cases where larger oblique angles both increase radiation output and put the patient's skin surface closer to the tube.^20^, ^21^, ^22^ If used as a radiation management strategy, tight collimation of the X‐ray beam increases the benefit of this technique.^20^ Onboard, real‐time PSD mapping capabilities, if available, can help determine the benefit of these strategies.

Until a time at which PSD is widely available as a real‐time displayed dose index, *K*_a,r_ is the preferred dose index for dose notifications in fluoroscopy. However, as PSD is more closely correlated with the likelihood of tissue reaction, it should be used for intra‐procedural notifications when available. Conservatively, values for both planes in biplane procedures should be summed for the purpose of intra‐procedural notifications.^1^ Multiple publications exist with a consensus recommendation for setting notification levels with the first notification at 3 Gy *K*_a,r_ and subsequent notifications every 1 Gy *K*_a,r_.^1^, ^2^, ^3^, ^4^, ^17^ However, there are practice‐specific considerations for setting notification levels, as well as patient‐specific considerations, such as the presence of sensitizing factors, that should be used to modify notification values when appropriate. Practice‐specific factors to consider when setting notification levels include:

1. *Types of procedures performed*: Depending on the procedure type (e.g., neuro, body, cardiac) the fluoroscopy dose notification level could be adjusted in consideration of the relationship between *K*_a,r_ and PSD. The geometry of the procedure affects the ratio of PSD to *K*_a,r_, denoted as the “dose index” by Miller et al.^4^ To avoid confusion, the “dose index” as described by Miller, will be referred to as the “PSD factor” in this report. For non‐isocentric procedures, such as those performed in vascular and interventional radiology, the PSD is often similar to the reported *K*_a,r_ (PSD factor ∼ 1.0).^23^ For isocentric procedures, where the relevant anatomy is placed at isocenter to facilitate the use of multiple gantry angles, the skin dose will be higher than the *K*_a,r_ if only a single projection, or a narrow range of projections, is used (PSD factor > 1.0). However, isocentric procedures often require the use of many different gantry angles, which may result in multiple distinct “fields” on the skin of the patient. This tends to reduce the dose index, and the PSD would be less than the *K*_a,r_ (PSD factor < 1.0).
2. *Equipment capabilities*: Many interventional fluoroscopes now offer the capability to program one or more fluoroscopy dose notification levels into the system. Upon reaching that notification level, an alarm sounds and/or a visual indication is displayed at the position of the operator. If, for example, a fluoroscope offers only three such levels at fixed values, this is a consideration when selecting the first, second, and third notification levels in policy. Different notification levels may be implemented for different services based on the type of procedures performed or the available dose indices. The QMP should ensure that any programmed dose notification levels are harmonized, though this can be complicated by differences in manufacturer's specific alert configurations.
3. *Alarm fatigue*: The 5‐min fluoroscopy timer is often ignored in interventional fluoroscopy because it sounds numerous times during most procedures. Avoiding similar alarm fatigue with dose notification levels is an important consideration, both for the value of the first notification level and the intervals between notification levels.

One other important intra‐procedural notification is the rapid recognition and correction of unsafe conditions when they exist. Examples of unsafe conditions include the presence of the patient's arm in the field of view when the arm is not the area of interest, and direct irradiation of the eye lens or female breast tissue when unnecessary.^24^ Every member of the procedural team should be confident in identifying such conditions when they exist and reporting them to the operator and responsible physician.

Diagnostic medical physicists can provide maximum value to their healthcare organizations only when they understand the clinical aspects of fluoroscopically guided procedures. Fluoroscopy dose indices can only be understood completely when accompanied by the clinical context of the procedure, and imaging protocols for fluoroscopy can be optimized only when the tasks involved in completing the procedure are understood. For these reasons, it is important that a QMP who supports a clinical FGI practice be provided clinical instruction and time to observe clinical procedures, including how the equipment and protocols are used by physician operators. It is likely that this observation time can also be used to optimize protocols.

### 5.4 Establishing the SRDL and post‐procedure actions

The substantial radiation dose level (SRDL) is an operational threshold for radiation dose above which additional post‐procedure actions for patient care should be taken due to the potential for biological harm. The SRDL should be set to a level such that a radiation dose below the SRDL is unlikely to result in a tissue injury for an average patient of normal radiation sensitivity. However, “there is no implication that a radiation level below an SRDL is absolutely safe or that a radiation level above an SRDL will always cause an injury,” and patient‐specific factors such as underlying conditions or medications could influence the threshold to induce a tissue reaction.^1^ Suggested values for the SRDL are 5 Gy *K*_a,r_ or 3 Gy PSD.^1^, ^17^, ^19^ Since real‐time PSD estimates are not typically available on most equipment, use of *K*_a,r_ is generally recommended to establish thresholds for post‐procedure follow‐up.

When setting the *K*_a,r_ SRDL value, the previously discussed PSD factor concept should be considered.^25^ If it is known that the entrance skin point is likely to be unchanged during a procedure or the skin is closer to the X‐ray source than the interventional reference point (IRP)/patient entrance reference point (PERP), a lower SRDL may be warranted.^14^ Similarly, for biplane procedures, the dose received from each plane should be added for SRDL purposes unless it is known that the fields do not overlap.

Each facility must have a policy for identifying patients who receive a radiation dose exceeding the SRDL and for providing patient management and follow‐up, including provision of post‐procedure information written in simple language. This document (see Appendix B, adapted from NCRP Report No. 168 language, for an example) should provide educational information about the procedure, follow‐up information, and provider contact information for questions or concerns. This information can be provided in discharge instructions, in the electronic medical record, or both. Determination of the individual responsible for distributing these instructions is vital and should be part of the facility's policy. Technologists, nurses, physicians, or physician assistants can be assigned this duty. Further, the facility must have an established system for patient follow‐up and must document any such communication in the medical record. Follow‐up may be in‐person or via telemedicine and may reasonably be provided by any trained clinical team member, under the direction of the performing physician. All tissue effects should be assumed radiogenic until proven otherwise. If a severe or prolonged radiogenic tissue reaction is observed, the patient should be seen in person by the performing physician whenever possible and referred to dermatology, wound care, radiation oncology, or another appropriate specialty for further care, with all appropriate information included in the patient's medical record.

PSD estimates may be useful for patient management in some cases. Facilities should have a defined process for requesting a PSD estimate performed by or under the supervision of a QMP. PSD estimates required by policy, regulation or accreditation standard, or requested from a licensed provider, must be documented in the patient medical record. The details of estimating PSD from dose indices are outside the scope of this document and have been described elsewhere.^26^, ^27^ Due to the various assumptions and inherent uncertainties involved, PSD estimates are unlikely to be better than ±50% accurate.^11^ This uncertainty should be included in any estimate and QMPs should always include a range of possible values in addition to the likely PSD value. For example, depending on various assumptions made regarding variable procedure aspects such as table height, collimation, and beam angulation, a PSD estimate could be documented as “likely 13 Gy, but with a probable range of 8–18 Gy.” As of January 2021, the American Medical Association (AMA) added a billable Current Procedural Terminology (CPT) code that refers to PSD estimates; code 76145 applies to evaluation of radiation exposure that exceeds institutional thresholds.^28^

### 5.5 Cumulative doses and risk of tissue reactions

There may be instances where a patient has multiple procedures in a short span of time where no single procedure reaches the threshold for patient follow‐up, but the summed doses warrant action. Setting an appropriate time window for a meaningful summation of doses is challenging. As previously mentioned, repair of sublethal radiation injury to skin is typically complete within a day of exposure, so doses delivered within a 24‐h period should invariably be summed. However, repopulation of damaged cells can require months. The SIR's guidelines for patient dose management suggest summing doses over a 60‐day period, while prior TJC sentinel event standards required summing doses over 6–12 months.^4^, ^17^, ^29^ This report recommends 60 days as the most biologically meaningful time window for summing patient skin dose. The same thresholds for follow‐up should be used for single or multiple cumulative procedures that exceed the SRDL.

Identifying patients with high doses from multiple procedures, and their potential risk, is technically challenging. The patient's history of radiation exposures may be incomplete if some exposures occurred at outside facilities, or even in a different department within the same facility, if procedure information is not shared. Even with sophisticated dose management software that can track patient dose, there can be a time lag in identifying these patients, who may be discharged before they are identified as having exceeded SRDLs. In these cases, facility policy should identify how the patient will be contacted and who will be responsible for follow‐up. The treating physician is ultimately responsible for ensuring follow‐up is performed.

## 6 SPECIAL CONSIDERATION FOR PEDIATRIC AND PREGNANT PATIENTS—QMP INVOLVEMENT IN RISK ASSESSMENT

As previously described, QMPs are generally the most knowledgeable individuals on radiation risk and play an important role in the fluoroscopic dose management process. This knowledge is critical in cases of pediatric or pregnant patients undergoing fluoroscopic procedures, as both can be at higher risk of radiation effects. On average, both the pediatric patient and the fetus are at higher risk for stochastic effects from radiation exposure due to more rapid cell growth and a comparatively greater number of undifferentiated cells compared with adults, as well as their longer remaining expected lifespan.^30^ In addition to these risks, each has the possibility of unique tissue reactions. Pediatric patients have shown a lower threshold for cataract development.^10^ High fetal doses during particular stages of development can result in loss of pregnancy, fetal organ malformation, or intellectual disability.^31^ In addition to the dose estimation roles discussed below, for both pediatric and pregnant patients, the QMP can play a role by providing direct counseling to the patient or guardian in order to provide information and address any potential radiation concerns.

The principal role of the QMP in pediatric fluoroscopy should be dose management in patients, ensuring that the radiation dose is no greater than necessary to maintain image quality adequate for the clinical task. QMPs should ensure that clinical staff are aware of the magnitude of the radiation risks involved and any thresholds for effects. The QMP must consult with clinical teams to ensure that appropriate fluoroscopic equipment is used and protocols are optimized for pediatric patients. This is particularly important, as patients can range in size from premature infants weighing less than 1 pound to adolescents who exceed normal adult dimensions. Examples of protocol elements that the QMP should consider, when appropriate, for pediatric fluoroscopy are:

1. Reduced pulse rate or width.
2. Reduced tube current.
3. Use of small focal spots for higher resolution imaging.
4. Removal of the anti‐scatter grid in smaller patients.
5. Selection of appropriate image‐processing parameters.
6. Selection of any other appropriate patient dose reduction controls.

Operators should be mindful of routine methods for dose reduction, such as proper attention to collimation, magnification, pulse rate, and source‐to‐skin and source‐to‐image‐receptor distance. Regular use of pre‐procedure checklists, such as the one provided by Image Gently, can help the QMP operationalize these behaviors.^32^

For fluoroscopy of a pregnant patient and fetus, the QMP plays a similar role. There may also be instances in which FGI procedures are performed on a woman with an unknown pregnancy. In these cases, the QMP will often be involved in estimating fetal dose. Details of fetal dose estimate methods can be found in Wagner et al.^31^

When fluoroscopic imaging is necessary on a known pregnant patient, clinicians should consult with the QMP to estimate the radiation dose and potential risk to the fetus. Procedure planning should include a consideration of imaging protocol optimization, collimation, and the relationship between fetal dose and displayed dose indices. Additionally, the QMP may be asked to be available during the procedure for consultation and to make sure adequate information is obtained for a fetal dose estimate. More specific fetal dose estimates can be obtained with the use of thermoluminescent dosimeter (TLDs) or Optically stimulated luminescence dosimeter (OSLDs), as described in Dauer et al.^33^ With appropriate preparation, complex interventional procedures in the abdomen and pelvis, such as renal or trauma embolizations, can often be completed with relatively low fetal doses.

## 7 FLUOROSCOPIC EQUIPMENT EVALUATIONS

Certain aspects of acceptance testing, commissioning, and periodic acceptability testing of fluoroscopy systems deal with patient and staff radiation dose management.^24^ Such aspects include, but are not limited to, scatter survey and comparison to manufacturer‐provided isokerma plots, measurement of table and pad transmission factors, measurement of typical and maximum air kerma rates for fluoroscopy and acquisition modes of operation, imaging protocol design and review, and measurement of the accuracy of machine‐reported dose indices. Accreditation and regulatory requirements may require additional tests and establish minimum standards and frequencies for such evaluations.^34^ AAPM Task Group reports, Medical Physics Practice Guidelines (MPPG), and American College of Radiology (ACR) Practice Parameters may provide guidance on test methods, expected values, and the role of the QMP and other personnel in performing these evaluations.^35^, ^36^, ^37^, ^38^, ^39^ It is also important to establish performance baselines during acceptance testing, compare these baselines to manufacturer specifications, where applicable, and to compare future measurement values to established baselines.

## 8 THE JOINT COMMISSION FLUOROSCOPY REQUIREMENTS

TJC, a healthcare accrediting body in the United States, has instituted standards on the use of fluoroscopy and on the management of radiation exposures to the patients of accredited facilities. Effective 1 January 2019, healthcare organizations accredited by TJC are required to meet new “elements of performance” related to fluoroscopy, including annual equipment performance evaluations, documentation of procedure dose indices, setting SRDLs, and review of procedures exceeding the SRDL.^34^, ^40^, ^41^, ^42^, ^43^ These requirements are in addition to TJC's updated fluoroscopy sentinel event, which requires root cause analysis review if permanent tissue injury results from improperly performed procedures.. The QMP is an integral part of the healthcare team tasked with meeting these requirements. TJC rescinded pre‐publication standards that specifically required annual radiation dose optimization training for fluoroscopy operators.

### 8.1 Annual equipment evaluation

TJC standards (Element of Performance No. 34, EC.02.04.03) state that at least annually a diagnostic medical physicist must conduct a performance evaluation of all fluoroscopic imaging equipment. This requirement excludes equipment used for therapeutic radiation treatment planning or delivery, but includes FGI suites, over‐ and under‐table fluoroscopy rooms, and mobile and mini C‐arm fluoroscopes.^34^ AAPM MPPG 10a suggests that all fluoroscopic performance evaluations be performed by a QMP, though according to TJC, they may be assisted by individuals with the required skills, as determined by the QMP.^34^, ^37^

### 8.2 Dose index documentation

TJC standard (Element of Performance #30, PC.02.01.01) states, “The reference‐air kerma, cumulative‐air kerma, or kerma‐area product are [sic] documented in a retrievable format … such as a picture archiving and communication system.” (Note that “reference air kerma” and “CAK” are two terms for the same dose index: *K*_a,r_.)^41^ If a system does not display the CAK or KAP, the fluoroscopy time, mode of operation, and number of images should be documented instead, in a retrievable format. According to a clarification from TJC in January 2019, this element of performance does not apply to fluoroscopy equipment used for therapeutic radiation treatment planning or fluoroscopy equipment classified as a mini C‐arm. Documentation is still required for other non‐FGI fluoroscopes such as full‐sized mobile C‐arms, remote, and tableside fluoroscopy systems, despite the fact that there can be considerable cost involved in implementing such a system and questionable benefit as the entrance skin dose resulting from these systems are typically very low.

A facility's choice of method for documenting fluoroscopy dose indices depends on the equipment in use, the size of the healthcare system, and the resources and technology available. Possible solutions include:

1. Archival of fluoroscope produced dose information into picture archiving and communication system (PACS).
2. Manual logs.
3. Manual entry into permanent patient records, such as patient electronic medical records (EMR), the PACS, and hospital or radiology information systems (HIS and RIS).
4. Automatic radiation dose index monitoring software.

The QMP, as a subject matter expert, is often involved in helping facilities comply with these requirements. These solutions require a collaborative approach between medical physics and hospital Information Technology (IT) and/or PACS administrators, and each facility needs to determine which approach is best, based on the capabilities of their equipment and available technology.

One solution to documenting dose index data is to rely on RDSRs and dose summary pages produced by the modality, which are often sent to PACS along with any procedure images. While this approach is appealing in that it is simple and requires no additional software systems or manual recording of data, it has several potential disadvantages. Procedure dose summaries and RDSRs are common on modern FGI equipment, but legacy, general, or mobile fluoroscopes might not produce them, necessitating additional documentation methods. The dose summary approach can also result in fragmented storage locations, as dose data reside within individual patient PACS records instead of a central location. Often, various hospital services utilize entirely separate PACS systems, which can further complicate data retrieval. Storage of dose index data in multiple locations can hinder the ability to aggregate dose indices from multiple procedures or to do facility dataset review for quality improvement purposes as discussed later in this report.

Paper logs are the simplest and least expensive to implement and meet the recommendations but present possible Health Insurance Portability and Accountability Act (HIPAA) issues due to protected health information (PHI) required for retrieval, as well as potential accuracy and legibility issues common to manual records. Additionally, paper records, if used, do not lend themselves easily to data analysis. Having dose index, equipment, and operator information in a digital format makes it possible to better evaluate dose indices and equipment use, in addition to allowing easier auditing of operators’ habits. For these reasons, this MPPG recommends paper logs be used for fluoroscopy dose index tracking only when digital formats are not possible. Manual entry into digital formats, such as spreadsheets, can avoid some potential HIPAA issues and can be better used for data analysis, but may still suffer accuracy issues from manual entry.

Fluoroscopy procedure information entered into digital formats has the advantage of being inherently electronic and can often be directly linked to sources such as HIS, RIS, or HL7 feeds, which can automatically populate patient information with high fidelity. This method also avoids potential HIPAA issues, as these systems are generally much more secure than physical notebooks or spreadsheets stored on network drives. Reports including patient and procedure information, equipment operator, and dose indices can be easily generated from these data, which can be useful for auditing and analysis, though manual entry can lead to data errors and false alarms. Specific methods of data entry can vary. Since fluoroscopy equipment is often operated outside of radiology departments, access to software systems should be considered. Data entry into systems such as a RIS may not be possible in hospital departments outside of Radiology, possibly necessitating multiple avenues of data entry for a centralized dose index database. The QMP will likely need to work closely with hospital IT and/or PACS administrators in order to set up such a system.

Automatic radiation dose index monitoring systems can eliminate most manual entry and automatically populate a database with patient and dose index information, greatly increasing data fidelity. This functionality is dependent on the fluoroscopy equipment in use. While most new FGI equipment can produce RDSRs that are sent directly from the modality, much of the fluoroscopy equipment used today does not support RDSR functionality and may not be integrated easily into commercial radiation dose index monitoring systems. Specifically, it may be difficult to get dose information from legacy equipment, R/F rooms, and mobile or mini C‐arms into these systems, necessitating a separate documentation system for those data. An additional challenge is connectivity between dose index monitoring systems and patient electronic medical records, leading to lack of access to patient dose data for referring physicians or interventionalists who perform FGI procedures. Better integration of these systems would improve patient care by providing immediately available radiation dose information to physicians.

Regardless of the specific method for storage, regular monitoring and maintenance of the radiation dose index database is critical to its long‐term success. New staff, new devices, and software upgrades have the potential to disrupt the methods used to document radiation dose indices. Even without changes to the hospital workflow, missing or incomplete data are possible. Regular system monitoring and maintenance allows for adjustments as needed to ensure that the quality of radiation dose data is maintained and documented. Again, the QMP is a likely candidate for this task, though time should be allocated for this work, which can take considerable effort. Most facilities will adopt an iterative approach and continue to improve their data capture process over time.

### 8.3 Identification of radiation exposure thresholds

TJC standard (Element of Performance #30, PC.02.01.01) requires a facility to “identify radiation exposure and skin dose threshold levels, that if exceeded, trigger further review and/or patient evaluation to assess for adverse radiation effects.”^42^ TJC does not provide any specific recommendations for threshold levels but does refer to NCRP Report No. 168. Prior discussion of SRDLs in this document addresses recommendations for complying with this standard. The QMP should be involved in setting these threshold levels and drafting policies.

### 8.4 Reviewing and analyzing procedures over threshold

TJC standard (Element of Performance #20, PI.02.01.01) requires that the organization providing fluoroscopy services “review and analyze instances where the radiation exposure and skin dose threshold levels identified by the organization are exceeded.”^43^ TJC does not limit this threshold review to FGI procedures but includes all fluoroscopic services. TJC does not specify how this review and analysis is to be accomplished, only that it be done. Previous discussion in this document regarding appropriate patient follow‐up for procedures above the SRDL is presumed to be sufficient. For policy purposes, the prior recommendations in this document may be followed with the FGI SRDL applied to lower dose fluoroscopic equipment as well (mobile C‐arm fluoroscopic units for use in operating rooms, general fluoroscopic units used for low‐dose diagnostic studies, etc.). Procedures completed with this equipment typically have very low *K*_a,r_ and it is unlikely that any non‐interventional procedure will have a *K*_a,r_ above the SRDL.

### 8.5 Sentinel event requirement

TJC adopted a sentinel event policy in 1996 for monitoring patient safety events that lead to death, permanent harm, or severe temporary harm, and that are not related to the natural course of a patient's underlying illness or condition.^44^ In 2005, TJC added “radiation overdose” as a reviewable sentinel event, which, in addition to the delivery of radiotherapy to the wrong region or >25% above the planned dose, included “Prolonged fluoroscopy with cumulative dose >1500 rads [15 Gy] to a single field.”^29^ In a subsequent publication, the TJC clarified that monitoring cumulative fluoroscopy PSD over a period of 6 months to 1 year would be reasonable, ultimately leaving the decision of determining the cumulative dose monitoring time window to the accredited institution.^29^ If a sentinel event occurs, TJC requires conducting a comprehensive systematic analysis, identifying causal and contributory factors, and documenting a corrective action plan, as well as a recommendation (but not requirement) to report the event to TJC.^44^ The most common approach to this analysis is a Root Cause Analysis (RCA), which needs to be completed within 45 days of becoming aware of the event. Additionally, in order to be considered a credible analysis, the RCA is required to include senior healthcare organization leadership.^44^

Practical implementation of the “radiation overdose” sentinel event has been difficult. Compliance required knowledge of PSD, an index not widely available on fluoroscopic equipment. For the majority of fluoroscopy systems that do not report PSD, estimations of patient and procedure‐specific PSD are laborious and prone to uncertainty. Even with all information available, skin dosimetry estimates are unlikely to be more accurate than ±50%.^11^ Due to PSD uncertainty and variation in individual radiosensitivity,^44^ it is possible for patients to develop severe tissue reactions at estimated PSDs below the 15 Gy sentinel event definition. Conversely, it is possible for patients with estimated PSD greater than 15 Gy to experience no tissue effects.

The “radiation overdose” fluoroscopy sentinel event was unique among TJC sentinel events. Other sentinel events include patient suicide, abduction, or elopement leading to death or serious harm, unanticipated death of an infant, discharge of an infant to the wrong family, wrong site or patient surgeries or radiotherapy, or assault or homicide of patients or staff.^44^ All of these can reasonably be described as preventable events that should *never occur* in the normal provision of healthcare services. While high radiation tissue doses can result in temporary or permanent harm, a PSD exceeding 15 Gy does not necessarily indicate that standards of care were not upheld. Some complicated FGI procedures require very large radiation skin doses to complete, even when justified and optimized. There is also wide variation in patient size and lesion characteristics for some FGI procedures, which may result in wide ranges of PSD. This is not to say that high skin doses should not be investigated, or that it is not possible that severe tissue reactions could, in some cases, have been avoided by better practice. However, investigating a “radiation overdose” sentinel event often led to large expenditures of time and resources in pursuit of RCA of properly performed procedures, and potentially excluded investigation of serious tissue reactions that occurred at a PSD below the rigidly defined PSD threshold.

Due to these limitations, TJC made changes to the fluoroscopy sentinel event requirements in 2021, with changes going into effect in January 2022. The updated requirement now defines a sentinel event as *“*Fluoroscopy resulting in permanent tissue injury when clinical and technical optimization were not implemented and/or recognized practice parameters were not followed.”

## 9 THE NCRP STATEMENT 11 PROCESS

NCRP Statement No. 11 provides an administrative process for facilities to manage certain adverse events from FGI procedures in line with the new TJC sentinel event definition, which triggers a review based on identified tissue reactions rather than a PSD threshold.^45^ Since tissue reactions can occur at PSD below the previous 15 Gy threshold, the Statement No. 11 process factors in an individual patient's radiosensitivity and applies to procedures that would not previously rise to the level of a sentinel event. By focusing on patient outcomes rather than PSD thresholds, the NCRP process also avoids the ambiguous time frame for multi‐procedure dose accumulation in the TJC's previous sentinel event definition. It also eliminates any ambiguity as to whether an estimated PSD value with large uncertainty requires a full RCA.

NCRP Statement No. 11 recommends that facilities develop a quality assurance and peer‐review (QA‐PR) program. This program promotes radiation management by tracking and reviewing available radiation dose indices periodically and triggering patient follow‐up when specified threshold values are exceeded, as previously discussed in this document. The program includes a QA‐PR committee, which evaluates radiation management for FGI procedures based on clinical and dosimetric data. This committee is composed of a QMP and professional practitioners so that it is competent to evaluate the clinical appropriateness and relevance of quality and safety matters. This committee would then suggest and implement corrective actions as needed.

In instances where patient follow‐up results in a suspected clinically important radiogenic tissue reaction, this QA‐PR committee would evaluate the procedure, including clinical and dosimetric data, to determine if it met recognized practice parameters, using the following criteria, where applicable:

1. Clinical justification of the procedure.
2. Proper pre‐procedural review and evaluation of the patient's past FGI encounters for skin injury.
3. Proper discussion of the potential for tissue reactions during informed consent process.
4. Appropriate use of radiation during the procedure.
5. Appropriate post‐procedure patient follow‐up.

According to the NCRP, the possible outcomes of the QA‐PR evaluation are:

1. The tissue reaction was detected through follow‐up and likely unavoidable. No action required.
2. Clinical or technical optimization might have reduced the severity or improved in the detection of the reaction, but overall practice criteria were met. Methods for optimization should be implemented.
3. Radiation use did not meet recognized practice parameters. A clinically important tissue reaction was potentially avoidable, its severity could have been minimized, or it was not detected. Corrective action is required.

Statement No. 11 recommends that an RCA be undertaken only in the last case, if one or more practice parameter criteria were not met. This initial QA‐PR review eliminates the need for a full RCA with executive administration involvement for procedures where appropriate clinical care was provided.

The initial goals of TJC's “radiation overdose” sentinel event were proper identification and follow‐up of patients with the potential for tissue effects, and the identification of instances where high tissue doses were not justified. It is the opinion of this group that these goals are better accomplished under the new sentinel event requirement and by following the recommendations of NCRP Statement No. 11, which makes better use of clinical resources to address TJC's concern of undiagnosed radiation‐induced tissue effects. Under this system, a PSD calculation by a QMP is only needed to direct proper dermatological care in cases of a known severe reaction, or as required by the facility's established process for PSD evaluation. Radiation fields should be assumed to be overlapping unless evidence suggests otherwise. Readers are again referred to Jones and Pasciak^26^ and AAPM TG 357 for more detailed discussion of performing PSD estimates.^27^ In cases where radiation use did not meet recognized practice parameters, an RCA, including the QMP and members of hospital executive administration, must be performed as described by TJC.^46^

## 10 SUGGESTED STATE REGULATIONS

The CRCPD maintains guidance that states may use when drafting regulations regarding the safe use of ionizing radiation through its Suggested State Regulations for Control of Radiation (SSRCR, or more commonly, SSR). The SSRs are adopted without modification by some states as regulations. The following discussion is based on Part F of the SSRs “Medical Diagnostic and Interventional X‐ray and Imaging Systems.”^47^

### 10.1 Radiation Protocol Committee

The SSRs recommend the creation of a Radiation Protocol Committee (RPC) responsible for ensuring “that exams being performed achieve the desired diagnostic image quality at the lowest radiation dose possible while properly exploiting the capabilities of the equipment being used.”

The SSRs state that the RPC includes, at a minimum, a supervising physician, a QMP, and a lead technologist. This MPPG recommends the following specific responsibilities for each of these individuals:

1. Supervising physician: The supervising physician, a physician who performs FGI procedures, has the responsibility for overseeing the activities of the RPC. This physician also works with the lead technologist to develop and maintain imaging protocols. When necessary, this physician serves as the liaison to other physicians for issues related to patient safety, such as long fluoroscopy times compared to peers.
2. QMP: The QMP brings a knowledge of radiation safety and dosimetry to the RPC. The QMP is responsible for testing, or overseeing testing, of the FGI equipment to ensure it operates safely. The QMP also provides PSD estimates when necessary and advises on the impact of the administered radiation dose on the patient.
3. Lead technologist: The lead technologist is responsible for maintaining imaging protocols under the direction of the supervising physician and QMP. This technologist advises the other RPC members of equipment issues or other observations that may affect patient dose or image quality. When PSD estimates are required, the lead technologist assists the QMP in gathering the necessary data.

The organizational structure of the healthcare system will dictate the optimal committee arrangement. For a stand‐alone facility, if different medical specialties utilize FGI equipment (e.g., interventional cardiology, interventional radiology, vascular surgery), the facility should consider including physicians and technologists from each specialty. Other possible committee members are department managers, health system risk managers, and the radiation safety officer (RSO). Other individuals may be added to the committee as deemed necessary. If a larger healthcare system has more than one facility, a system‐wide committee may be established to ensure consistency among facilities. In this structure, one RPC may be formed, provided each facility within the enterprise has appropriate representation on the committee. Another option is to add the scope of the RPC into the responsibilities of an already established Radiation Safety Committee, as long as the recommended RPC membership is met.

Per the SSRs, the RPC is responsible for establishing procedures and protocols to be followed before, during, and after FGI procedures. The SSRs state that protocols addressing the following are reviewed by the committee at least annually:

1. Authorized users of FGI equipment.
2. Intra‐procedure patient radiation dose monitoring.
3. Dose notification levels.
4. Establishment of SRDL values.
5. Actions to be taken when a SRDL is exceeded.

These protocols are discussed elsewhere in this report. The SSRs only require the RPC to create and implement policies and does not require committee oversight of clinical data. However, given the membership, it is the recommendation of this MPPG that such review fall under RPC oversight. This review includes evaluation of clinical and dosimetric data from any procedure in which the SRDL is exceeded or a tissue reaction has occurred, as well as analysis of facility or system case volume and dose index datasets, described in a later section. These datasets can include case volume and comparative dosimetry. The duties of the RPC could also be extended to include the previously discussed QA‐PR functions.^45^ In doing so, the committee reviews procedure justification, patient‐specific factors, radiation dose optimization, the time course over which radiation doses were administered, disease severity, and procedure complexity.^45^ Depending upon the size of the healthcare system, initial oversight might better be accomplished by department‐level committees. Under such a framework, each individual department (e.g., interventional cardiology, interventional radiology, vascular surgery), would have its own committee to oversee clinical and dosimetric data, and to perform QA‐PR review of the clinical appropriateness of procedures resulting in tissue effects. The results of this department‐level review would then be sent to the facility or system‐wide RPC for final review and approval.

The healthcare system determines the frequency of RPC meetings. The SSRs suggest that the committee meet at least annually. More frequent meetings may allow for more rapid identification of potential problems and swifter implementation of subsequent changes in practice. Considerations that affect meeting frequency include the duties of the committee, the volume and complexity of FGI procedures performed, the number of physicians and medical staff involved, and the capabilities of the imaging equipment. Of these, procedure volume and complexity may be the most important, as both play a role in the number of potential procedures that may require committee review, if such review is tasked to the RPC. Some events may prompt the need for ad hoc meetings, such as sentinel events or serious patient injury review, per the previous NCRP Statement 11 QA‐PR discussion.^45^

The SSRs suggest that the RPC provide an annual report to the radiation safety committee, or, if the facility does not have a radiation safety committee, to the RSO. Although not addressed in the SSRs, this MPPG recommends that the annual report contain the following:

1. A list of individuals authorized to use fluoroscopic equipment for FGI procedures.
2. The total number of procedures that exceeded the SRDL and, if available, percentage of total procedures.
3. A review of all policies and procedures and any significant changes.

### 10.2 Procedures for maintaining records

The SSRs also recommend that all available radiation dose indices be recorded to facilitate skin dose estimation, if needed.

These recommendations are similar to TJC requirements previously discussed, and the role of the QMP is to provide guidance on what specific information must be recorded for skin dose estimates. If RDSRs are produced by a fluoroscope, they should be archived, if possible, and the QMP must have access to them when needed. Where the RSDR is not available, the QMP may request that additional information be recorded, such as gantry angles, fluoroscopic technique factors, table height, patient dimensions, etc. Appropriate patient follow‐up procedures, including skin dose estimation, are performed according to policies set forth by the committee.

### 10.3 Facility dataset review for quality improvement

Beyond CRCPD recommendations and TJC standards to review and analyze procedures over threshold values, collection of fluoroscopic dose index data provides facilities with quality improvement opportunities, as these data can be used to evaluate clinical practice. This type of analysis is useful because dose to the patient in fluoroscopy is highly dependent on the operator of the equipment. Since the QMP often oversees the collection of fluoroscopic data, they are well positioned to handle the task of data analysis. As detailed in NCRP Report 168, these data can be used to build “facility datasets” for each type of exam performed in a practice that can be compared among sites or to normative datasets, such as the American College of Radiology Fluoroscopy Dose Index Registry (DIR).^1^ These analyses can provide valuable insight into how the equipment is being used clinically and identify deviations from expected practice. NCRP Report No. 168 also recommends calculating the percentages of each procedure that exceed the SRDL set by the facility.^1^ This process can help determine those procedures that are more likely to result in high doses to patients. This information has the potential to affect the pre‐procedure consent process and room assignment decisions. Fluoroscopy dose index data may also be used to set procedure‐specific dose index review levels for evaluating clinical practice. These are not to be confused with the SRDL, which is a biologically based threshold related to the potential for tissue effects. Procedure‐specific review levels represent target achievable dose indices for specific procedures and can be useful for comparing clinical practice.

Beyond reported indices, collected data can be used to derive additional metrics for practice insight. Dividing *K*_a,r_ by total fluoroscopy time yields a measure of radiation utilization efficiency for a particular procedure, assuming fluoroscopy time is in some way related to the complexity of the procedure. For two procedures of similar complexity and fluoroscopy time, a procedure with higher utilization of acquisition or high‐level control modes would yield a higher value. Additionally, total *P*_KA_ and *K*_a,r_ data can be used to calculate the average field size (*P*_KA_/*K*_a,r_) at the IRP/PERP for a given procedure, thus permitting comparison of an operator's use of collimation. Beyond individual procedures, these derived metrics can be calculated for a fluoroscope or operator over a given month or quarter to assess and compare longer term operational trends. Values of any metric found to be substantially different from facility benchmarks can be investigated for potential protocol optimization or practice review. QMPs need to be mindful of the units used for a given dose index when comparing data. While *K*_a,r_ is often reported in units of mGy, displayed *P*_KA_ values vary by manufacturer and even by model, necessitating unit conversion for valid comparisons.

However, differences in procedural dose index data do not necessarily indicate improper practice. It is likely that more experienced physicians perform a larger share of more complex procedures, which would affect their data. A similar situation can arise with equipment, where a newer Interventional Radiology (IR) suite with more features is rightfully utilized more often for complex procedures than older equipment. Additionally, patient body habitus and anatomy can drastically affect dose indices. Individual procedures may exceed SRDLs not because they were performed improperly, but because larger patients or complicated vascular anatomy require higher tube outputs or longer procedure time.

The results of this type of analysis can be presented to operators or department chairs on a regular basis, either through a fluoroscopic RPC or dose review committee, or through other means such as existing staff meetings, radiation safety committee meetings, QA‐PR committee, or morbidity and mortality (M&M) conferences. This feedback can be an important part of practice improvement. Beyond comparing data within a practice, comparison to outside practices may be possible through programs such as the ACR Fluoroscopy DIR. The ACR Fluoroscopy DIR is the latest addition to the National Radiology Data Registry, and the first modality to join computed tomography (CT) in the DIR. The Fluoroscopy DIR provides a continuously updated normative dataset for fluoroscopically guided procedures, which can be used as an advisory dataset to which participating sites can compare their facility datasets of fluoroscopy dose indices.

The ability of a hospital to perform this kind of analysis will be heavily dependent on the resources available. If full procedure records, including the exam type, fluoroscope used for the procedure, operator, and available dose indices are electronically captured, datasets can be created and evaluated with relative ease. However, this level of investigation is not practical if paper logs are used to meet documentation requirements. If resources are limited, analysis should focus on higher dose FGI procedures, as they present the greatest radiation risk. However, if fluoroscopy data for all equipment are being collected electronically, further evaluations for basic fluoroscopy rooms and mobile C‐arms and their operators can be performed without significant additional effort.

## 11 ADDITIONAL FGI RELATED QMP DUTIES

The following sections briefly discuss QMP fluoroscopy duties beyond patient dose management and serve to direct the reader to related resources.

### 11.1 Training and privileging of fluoroscopy users

Training in the safe use of fluoroscopy, credentialing, and privileging of fluoroscopy users are important aspects of patient and staff radiation safety. Privileges identify which medical procedures a staff member may perform, while credentialing involves gathering relevant data for privileging an applicant. The ACR and AAPM recommend that each facility have a policy for granting privileges for fluoroscopy use, and CRCPD SSRs suggest this policy be developed by the RPC.^19^, ^47^ The QMP should be involved in the following tasks as they apply to the training and credentialing of fluoroscopy users: determining the need for a credentialing program, developing a fluoroscopy privileging policy, verifying compliance with requirements, developing and maintaining didactic content, and providing in‐person training on the safe use of fluoroscopy equipment. The nature of the training may be dictated by the specific scenario (e.g., new FGI operator vs. renewal, or FGI training vs. mini C‐arm training) as well as state regulations. Specific details of the QMP's role are beyond the scope of this practice guideline, but a comprehensive review is provided in AAPM Report No. 124.^48^

### 11.2 Occupational dose monitoring and badging

Personnel involved in interventional fluoroscopy procedures often require occupational radiation monitoring according to state regulations. For a given healthcare system, oversight of occupational dosimetry may be the responsibility of the QMP or a separate RSO. Even if not a direct job duty, the QMP may have better insights into clinical FGI practice and can aid in setting up a dosimetry program and can help verify the following:

#### 11.2.1 Appropriate personnel are monitored

Most state regulations mirror the SSR language that require monitoring of anyone likely to exceed 10% of the annual occupational dose limits.^49^ In an FGI environment, operators, be they physicians or technologists, generally require monitoring as they are likely to exceed the 5 mSv (500 mrem) effective dose equivalent (EDE) threshold, which is 10% of the annual (EDE) limit of 50 mSv (5000 mrem). However, depending on local practice and positioning during procedures, ancillary personnel such as nurses or anesthetists often record annual doses well below the 5 mSv threshold for monitoring. Ancillary personnel are often positioned farther from the patient during fluoroscopy and can be behind additional protective measures such as rolling shields. Previous dosimetry histories must be investigated in order to determine which personnel groups require monitoring. Monitoring policies may need to be reevaluated when practices or volumes change.

#### 11.2.2 Monitoring devices are appropriate

Dosimetry service providers must be accredited by the National Voluntary Laboratory Accreditation Program (NVLAP) for the type of radiation for which monitoring is performed. A one‐ or two‐badge protocol can be used to monitor occupational dose. Single monitoring devices are worn at the neck/collar outside any radiation personal protective equipment (PPE). The readings from this badge are directly used to calculate the various personal dose equivalents. Some states allow modification of measured deep dose equivalent according to equations in the SSRs and in NCRP Report Nos. 122 and 168 to account for the presence of PPE to derive a more accurate EDE (*H*_E_).^49^ This modification results in lower annual *H*_E_ readings compared to an un‐modified deep dose, which can be advantageous for workers approaching annual limits. The QMP must verify if this correction is allowed, as state regulations vary, and sometimes the correction can only be used in cases where the reported dose exceeds 25% of annual limits.

With a two‐badge protocol, a second monitoring device is issued and worn underneath the protective garment at the waist. A two‐badge protocol best represents the occupational dose to the worker and will result in lower *H*_E_ values than a corrected single‐badge protocol, though again, this may not be allowed by local regulation. Even when allowed, the two‐badge protocol has the disadvantage of requiring twice as many badges. In addition to greater cost and administrative burden, the two‐badge protocol increases the risk of lost or improperly used badges. Wearers often inappropriately mix up the collar and waist badges, leading to faulty data. A recent large scale review of occupational dose data from FGI areas noted that over a third of individual entries from two‐badge wearers were invalid due to either improper badge use, or failure to return both badges.^50^ Due to these likely operational problems with double badging, this MPPG recommends that a single‐badge protocol be used in FGI environments, with modifications to badge readings applied to account for the presence of leaded PPE whenever possible.

Doses to the extremities and skin (shallow dose equivalent) and lens of the eye (lens dose equivalent) are estimated by the dosimetry service provider based on the dose received by the monitoring device worn at the neck. Some dosimetry providers allow for corrections to lens dose to account for the use of leaded eyewear. Some also provide separate monitors that attach to eyeglass. Lens dose should be assessed, as evidence has shown a lower threshold for radiation‐induced cataract than previously thought.^51^, ^52^ The ICRP has lowered the annual dose limit to the lens to 20 mSv/year, compared to the 150 mSv limit in place in the United States. The NCRP has recommended a 50 mSv annual limit.^53^ The previous large scale study found that over 15% of workers exceeded the ICRP limit to the lens.^50^ The study found no difference in reported eye dose between a one‐ or two‐badge protocol.

#### 11.2.3 Monitoring frequency is appropriate

Any state regulatory requirements regarding monitoring frequency must be followed. Monthly monitoring is recommended as it permits more timely identification and investigation of high doses than does bimonthly or quarterly monitoring. However, less frequent monitoring may be appropriate for groups with lower occupational doses to reduce costs and administrative burden.

#### 11.2.4 Monitoring devices are used correctly by staff

Each individual should wear their assigned monitoring device(s) during procedures, at the correct location on their body and in the correct position with respect to the protective apron. When not in use, monitoring devices are stored in a location where they are not exposed to radiation above background levels. Control badges should not be exposed to radiation above background levels. Monitoring devices are exchanged, or data are retrieved from electronic monitoring devices in a timely manner. During the previously described observations of clinical FGI procedures, the QMP can observe occupational dosimetry usage and offer suggestions for improvement.

#### 11.2.5 Monitoring results are reviewed

Doses received by monitored employees should be reviewed at least quarterly to identify exposures above the facility's As Low as Reasonably Achievable (ALARA) investigational levels and regulatory limits. Local regulations and accreditation body requirements must be followed. Regular dose review can identify trends, opportunities for dose reduction, and possible non‐compliance issues relating to badge misuse or nonuse. Feedback to users, department chairs, and the RPC on the results of dosimetry review are an important part of quality improvement. The review may be done by the QMP, the RSO, or an individual delegated this responsibility by the QMP or RSO. Even in circumstances where an RSO oversees monitoring results, the QMP can provide additional radiation protection guidance to FGI participants though didactic or hands on demonstrations.

#### 11.2.6 Occupational radiation exposures meet regulatory requirements

Results should be compared to limits in state regulations, the US Nuclear Regulatory Commission (NRC) regulations, or, if applicable, United States Occupational Safety and Health Administration (OSHA) limits.^54^, ^55^ Mandatory reporting to applicable regulatory bodies must be performed if dose limits are exceeded.

### 11.3 Institutional review board

QMPs in academic or research settings may be tasked with participation in hospital institutional review boards (IRB), which oversee human research studies and ensure ethical treatment of participants. Most often, the QMP serves to provide dose and risk estimates for imaging research studies that differ from standard of care. NCRP Report No. 185, “Evaluating and Communicating Radiation Risks for Studies Involving Human Subjects: Guidance for Researchers and Institutional Review Board” provides useful information for QMPs serving this capacity.^18^

## 12 CONCLUSION

QMPs have a major role in helping healthcare institutions manage modern FGI practices. Their expertise related to the equipment, radiation biology, and regulatory environment surrounding fluoroscopy makes them an invaluable member of clinical team. QMPs can help in the development and implementation of appropriate protocols for managing patient radiation dose before, during, and after potentially high‐dose FGI procedures, as well as oversee analysis of clinical dose index data to further improve practice. Through educational efforts, QMPs can ensure that all clinical staff are appropriately informed of the potential radiation risks from fluoroscopic procedures so that appropriate measures can be taken to minimize risks and maximize benefits for patients and staff.

## AUTHOR CONTRIBUTIONS

This guideline was reviewed and updated by the Medical Physics Practice Guideline Task Group 343 of the Professional Council of the AAPM. Each author reviewed recent literature on the topic and offered opinions on and language for the guideline. They also reviewed and applied comments from the full AAPM membership to the document.

## CONFLICTS OF INTEREST

Jaydev K. Dave, PhD has received support from Philips Healthcare, GE Healthcare, and Lantheus Medical Imaging Inc. for research unrelated to this project. Philips Healthcare and GE Healthcare manufacture fluoroscopy equipment. Jaydev K. Dave has also provided imaging physics consulting services as a part of Rayscan Inc. A. Kyle Jones, PhD, FAAPM is President of FluoroSafety, a company that produces CME on quality and safety in medical imaging. Dr. Jones recused himself from all discussions related to personnel education and training during the development and review of this MPPG. The remaining authors have no conflict of interest.

## APPENDIX A

### High‐dose fluoroscopy sample consent language

You have been scheduled for a procedure that uses X‐rays, which are a type of radiation. Your doctor believes that the benefits of this procedure exceed the very small risks that may be associated with this use of radiation.

Skin changes like redness and hair loss are examples of the possible risks from radiation. These usually go away in a few days. In rare cases, they may be more severe. The risk of these skin changes depends on how much radiation is used.

You or your family will be told after the exam if skin changes are likely. If this happens, you will be given more instructions. These will tell you how to check your skin and what you should do if you see any changes.

## APPENDIX B

### High‐dose fluoroscopy discharge instructions

The procedure you had was done with X‐rays. X‐rays are a type of radiation. Because the procedure was complex, it required the use of more X‐rays than other types of imaging, such as a chest X‐ray or CT scan.

There is a chance that the X‐rays could cause skin changes in the area that was treated. These skin changes usually go away within a few days. Rarely, the skin changes can be more severe.

Over the next month, please check your skin in the treated area indicated above and watch for any of the following:

1. A red area, about the size of your hand.
2. Flaking skin, like a sunburn.
3. Small areas of hair loss.
4. Constant itching in the same area.

If you see any of these signs, please contact us as soon as possible to determine if any treatment is needed, or if the changes will get better without treatment.

____________________________________

Name

____________________________________

Phone Number

____________________________________

Email Address
